# Supplementary material for: Causal Relationship between Aging and Anorexia Nervosa: A White-Matter-Microstructure-Mediated Mendelian Randomization Analysis
Source: Biomedicines. 2024 Aug 16;12(8):1874. doi: 10.3390/biomedicines12081874 (PMC11351342; doi:10.3390/biomedicines12081874)
Supplement: Supplementary file 1 [file biomedicines-12-01874-s001.zip › Supplementary Figure/Figure S8/Leaveoneout plot of MR analysis for TL to ubm-b-1577.pdf]

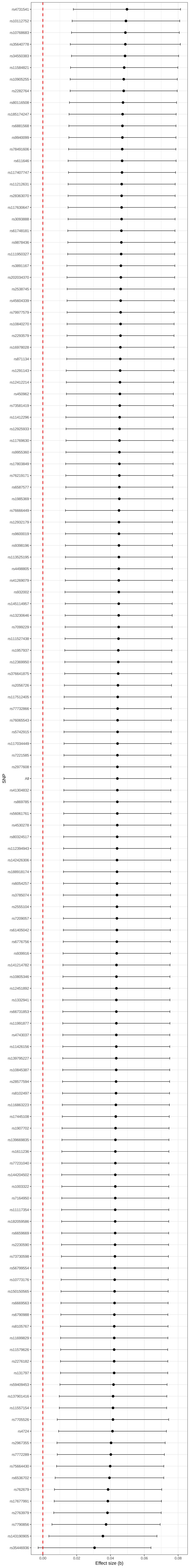
rs4731541
rs10112752
rs10768683
rs35640778
rs34550383
rs11584821
rs10905255
rs2282764
rs80116508
rs185174247
rs6881568
rs9940099
rs78491606
rs611646
rs117407747
rs11212631
rs117630647
rs28363070
rs3093888
rs61748181
rs9878436
rs111950327
rs3891167
rs202034370
rs2538745
rs45604339
rs79977579
rs10840270
rs2293579
rs16978028
rs871134
rs1291143
rs12412214
rs450962
rs73581419
rs11412296
rs12925933
rs11769630
rs9955360
rs17803849
rs76219171
rs6587577
rs1985369
rs76666449
rs12932179
rs9600019
rs9398196
rs113525195
rs4498805
rs41269079
rs932002
rs145114957
rs13230646
rs7099229
rs111527438
rs1957937
rs12369950
rs376641875
rs2056726
rs117512405
rs77732866
rs76065543
rs5742915
rs117034449
rs7221585
rs2977608
All
rs41304832
rs869785
rs56061761
rs4530278
rs80324517
rs112394943
rs142426306
rs189918174
rs6054257
rs3785074
rs2555104
rs7209057
rs61405042
rs6776756
rs939916
rs141214782
rs10805346
rs12451892
rs1332941
rs66731853
rs11991877
rs4743037
rs11426156
rs139795227
rs10845387
rs28577594
rs8102497
rs116863223
rs17445108
rs1907702
rs139669835
rs1611236
rs77231040
rs144204502
rs1003322
rs7164950
rs11117354
rs182059586
rs6659669
rs2230590
rs73730598
rs56799554
rs10773176
rs150150565
rs6669563
rs6790988
rs8105767
rs11609829
rs11579626
rs2276182
rs131797
rs59409453
rs137901416
rs11557154
rs7705526
rs4724
rs2967355
rs7772289
rs75664430
rs6536702
rs762679
rs17677991
rs2763979
rs7790856
rs143190905
rs35446936
SNP
0.00
0.02
0.04
0.06
0.08
Effect size (b)
